# A conservative approach to localize loose implant screw through cemented crown: an in vitro experimental study

**DOI:** 10.1186/s12903-024-04369-5

**Published:** 2024-05-28

**Authors:** Kale Masoud Mohammad Saeed, Abdulsalam Rasheed Al-Zahawi

**Affiliations:** Department of Conservative Dentistry, College of Dentistry, University of Sulaimani, Sulaimaniyah, 46001 Iraq

**Keywords:** Conservative approach, Loose implant, Access hole, Zirconia, Ceramics fusion

## Abstract

**Background:**

Retrieval of cement-retained implant-supported restorations is intriguing in cases of screw loosening. Detecting the estimated size of the screw access hole (SAH) could decrease destruction to the prosthesis and preserve the crown.

**Objectives:**

To precisely localize loose implant screws through cemented crowns to reduce crown damage after screw loosening.

**Materials and methods:**

In this in vitro study, 60 cement-retained implants supported 30 zirconia-based, and 30 ceramics fused to metal (CFM) lower molar crowns were invented, and each was subdivided into three subgroups (10 each). In group I (AI/BI) (control), SAH was created with the aid of orthopantomography (OPG). In contrast, in group II (zirconia-crown), SAH was created with the aid of CBCT + 3D printed surgical guide with a 2 mm metal sleeve in subgroups IIA/IIIA and CBCT + MAR was used to develop SAH in subgroups IIB/IIIB. SEM and Micro-CT scanned the SAH openings to determine the diameter of the hole, cracking, chipping, and chipping volume.

**Results:**

Regarding the effect of plane CBCT and CBCT + MAR on prepared crowns, a highly significant association between group I with group II (*p* = 0.001) and group III (*p* = 0.002) was detected. Regarding the cracking of SAH, significant differences between the zirconium crown and CFM restoration (*p* = 0.009) were found, while for the chipping, no significant association was seen between groups (*p* = 0.19).

**Conclusions:**

CBCT, either as a plane CBCT or with MAR, significantly improved the accuracy of drilling the screw channel and decreased injury to the existing restoration and abutment, aiding in better localization of SAH in loosened implant abutment screws.

## Introduction

According to Brånemark, 1983 osseointegrated dental implants cover the treatment choices for patients experiencing prosthodontics [1]. A dental implant is recommended for edentulous patients due to its great success [2], especially for single-tooth restorations [3].

Cement- or screw-retained single-tooth implant is a prosthetic modality with significant advantages over conventional techniques [4]. The clinical situation determines the selection for screw or cement retention [5]. Cemented restoration has superior esthetic aspects, passive fit, low fabrication cost and simple with accurate occlusal morphology; however, it has severe biological complications, and it is hard to recover the restoration in screw loosening, ceramic chipping, or assessing the peri-implant tissue [5].

Possible problems of implantation are failure of osseointegration failure, surgical issues, peripheral bone loss, peri-implantitis, and esthetic, masticatory, and phonetic difficulties [6]. Additionally, screw loosening/fractures, implant/porcelain fractures, and preservation loss of implant-retained overdentures can also be observed [7]. Retrievability is challenging with cement-retained implant-supported prostheses, especially after porcelain fracture or screw loosening [8]. Abutment-screw loosening is a problem in cement-retained implant-supported restorations that might lead to mastication discomfort with screw fracture [9]. Frequently, the abutment screw becomes loose from the implant body [10] while the crown stays cemented to the abutment [11].

Various methods are designated to start the perforation position of cement-retained prostheses and place the abutment screw [12].

Clinical photographs during prosthesis placement, the coloring of the veneering porcelain or the utilization of diverse supervisory tools might help find the situation of the abutment screw [13]. Cone beam computed tomography (CBCT) to decide implant inclination has been validated [14], and it is widely available for dental applications [15]. CBCT can provide images in any desired plane, showing high-contrast structures [16]. CBCT is more susceptible to artefacts due to its small amount of radiation and cone-shaped X-ray source [17]. In dentistry, different types of metallic materials, like dental implants, gold, and porcelain-fused metal, have been applied for restorations [18].

CBCT devices with metal artefact reduction (MAR) have been established [19]. The MAR is used in image reconstruction and decreases image distortion, such as darkened regions caused by losses of grey values and bright streak artefacts [5]. Micro-CT systems perform different investigations, including educational purposes [20] and nondestructive analysis [21], and do not require specimen preparation, staining and slicing [22].

Thus, this study was designed to compare the effect of CBCT alone or its combination with MAR on the localization of the screw of the implant. It was also using these tools for guiding the precise location of the implant abutment screw hole.

## Materials and methods

### Study design

**Fig. 1:**
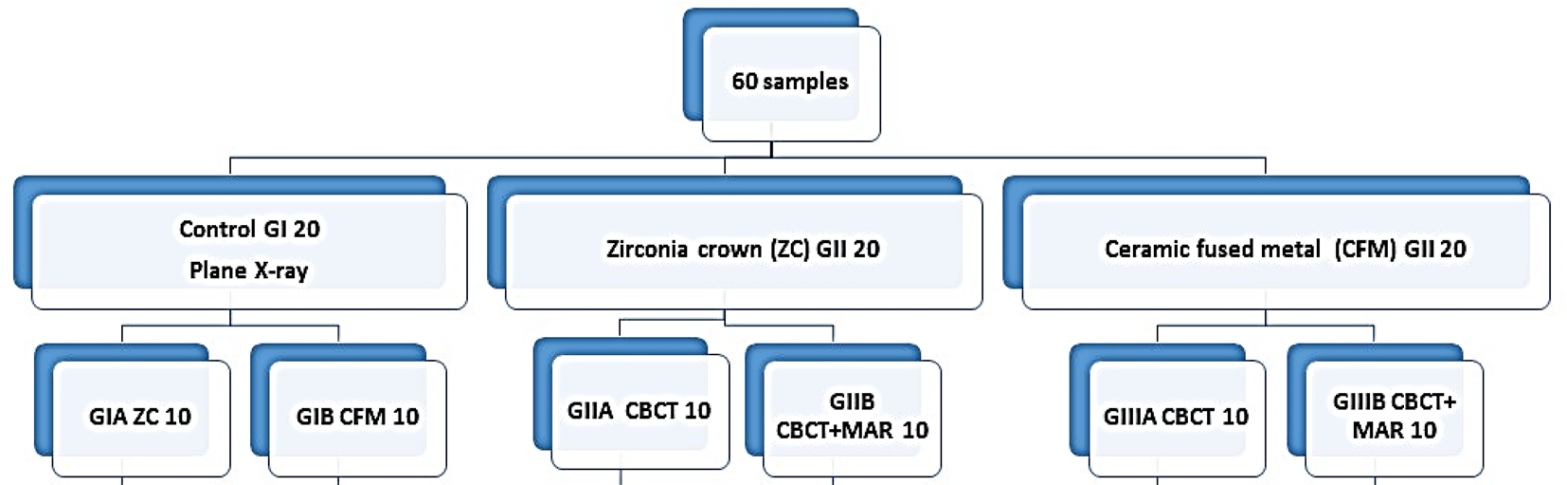
Study design. CBCT: Cone beam computed tomography; MAR: Metal artifact reduction

In this study, 60 cement-retained implants comprising 30 zirconia-based and 30 CFM lower molar crowns were invented, and each was subdivided into three subgroups (10 each) (Fig. 1).

### Preparation of the acrylic model

**Fig. 2:**
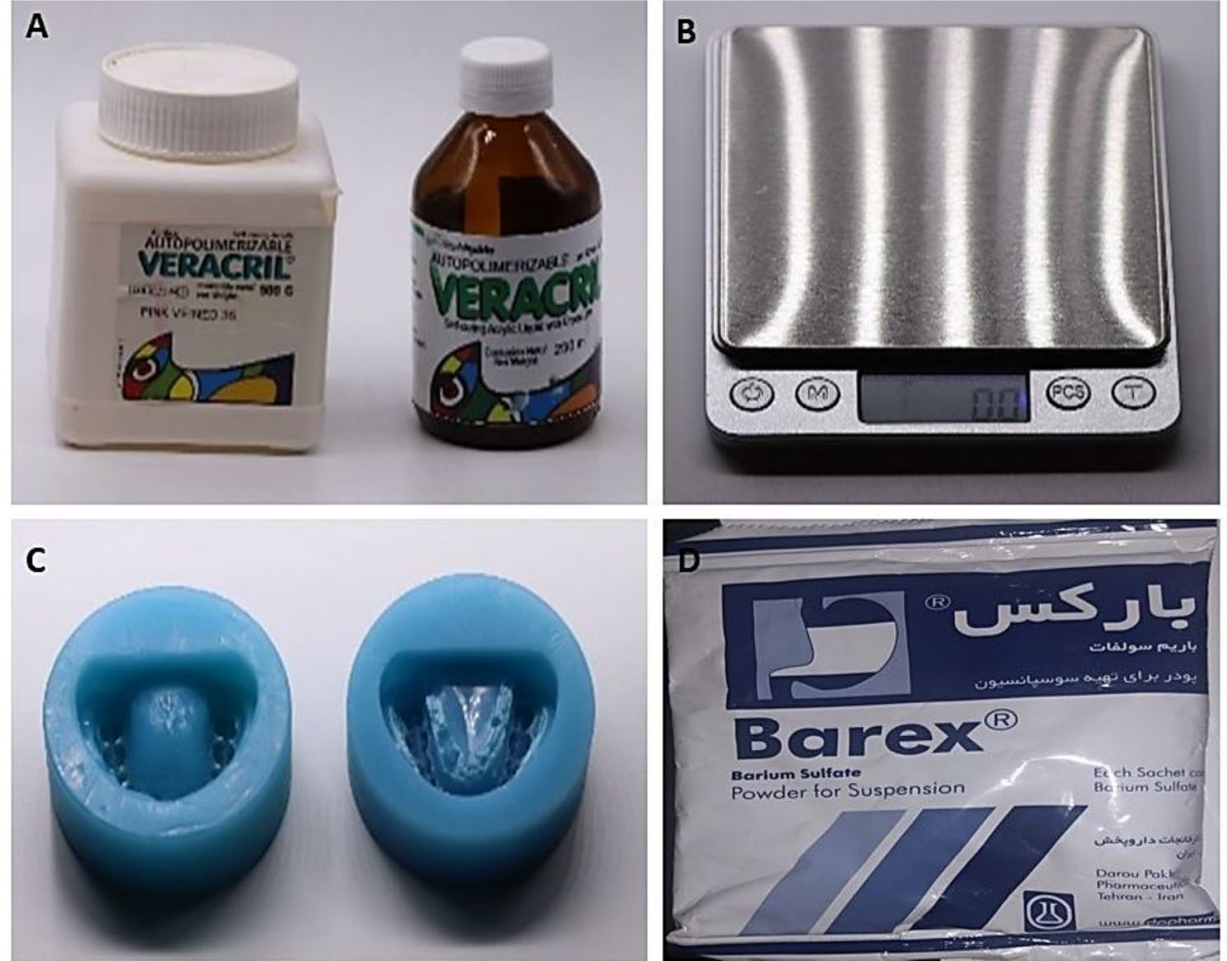
Materials for Cast construction indicating Veracril (**A**), sensitive balance (**B**), mold (**C**), and Barium sulfate (**D**)

A dental rubber mould (Buyamag, China) was used to duplicate the acrylic model for implant insertion at the lower first molars (right and left), which were blocked with wax. To create a model that could be seen by X-ray, barium sulfate powder (Barex, Italy) at concentrations of 40%, 30%, 20%, 10%, and 5% were added to the self-curing pink veined acrylic resin (Autopolimerizable-Veracril). Then, an OPG was taken for each prepared model to detect which barium sulfate concentration could show the acrylic transparency’s proper appearance. Adding 5% barium sulfate showed acceptable transparency of acrylic that approximated the spongy bone transparency and density when compared with mandibular bone by X-ray. Thus, 30 acrylic models were prepared by adding 5% barium sulfate powder, mixing it with the acrylic, pouring it into the mould, and leaving it until completely set. Lower first molars were removed from all models by lab straight handpieces and acrylic burs (Fig. 2).

### Implant insertions

After the construction of acrylic models, two system implants (4.5 mm × 10 mm) (TSA SA fixture No Mount, Korea) were inserted at the lower first molar (left and right) by an expert dentist in dental implantology. Osteotomies were performed on each acrylic resin model with surgical burs from an implantation surgery kit, and then 60 implants were inserted.

### Crown Construction

**Fig. 3:**
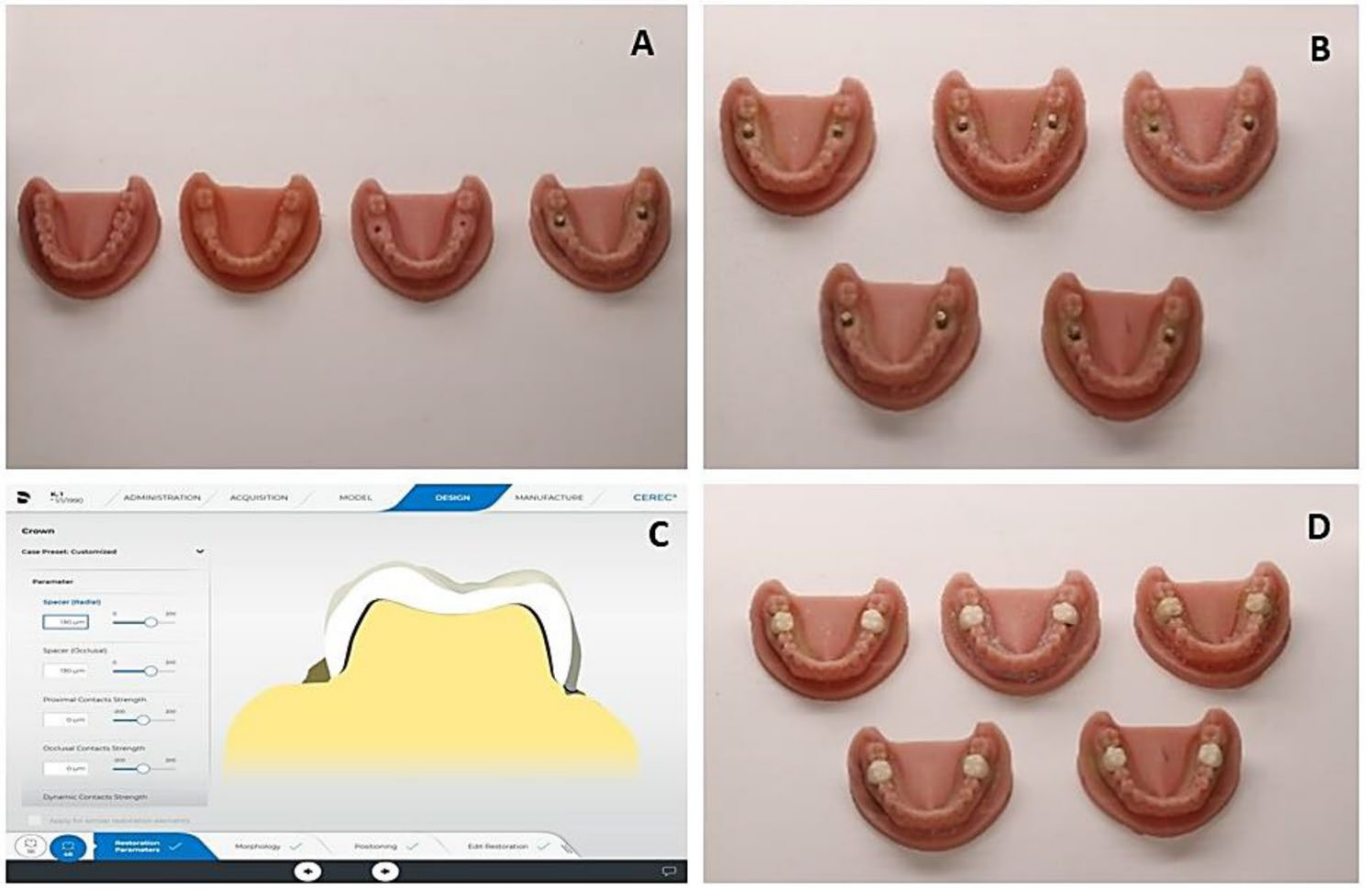
Construction of crowns. (**A**) Prepared implant, (**B**) Inserted implant, (**C**) Process of designing, and (**D**) Ready implants and crowns

After attaching the implant scan body to the inserted fixture, all models were scanned with a CEREC chair-side Omnicom scanner. The CAD/CAM software (Cerec System Ans Cerec SW 5, 4, and 2) was used to design monolithic zirconia crowns with cemented space of 60 μm × 1.5 occlusal, and radial thickness was applied for all crown designs. The designed crowns were sent to the dental milling machine (Sirona Cerec MC XL), and 30 monolithic crowns were manufactured using the CAD/CAM CEREC zirconia block. All milled crowns (CEREC MC XL Dentsply Sirona) were sent to the speed fire furnace (CEREC Speed Fire-Dentsply Sirona) for crystallization. The scanned files were sent as an STL file to the laboratory to design another 30 CFM crowns (Fig. 3).

### Implant cementation

Following the manufacturer’s instruction, each fabricated crown was cemented using glass ionomer cement/Gold Label (GC Corporation, Japan). Consequently, the abutment screw hole was filled with pieces of guttapercha. The zirconium and CFM crown groups were divided randomly into three groups (no = 20 in each), and each group included ten zirconia crowns and 10 CFM crowns.

### Detection of implant direction by X-ray, CBCT, and CBCT + MAR

**Fig. 4:**
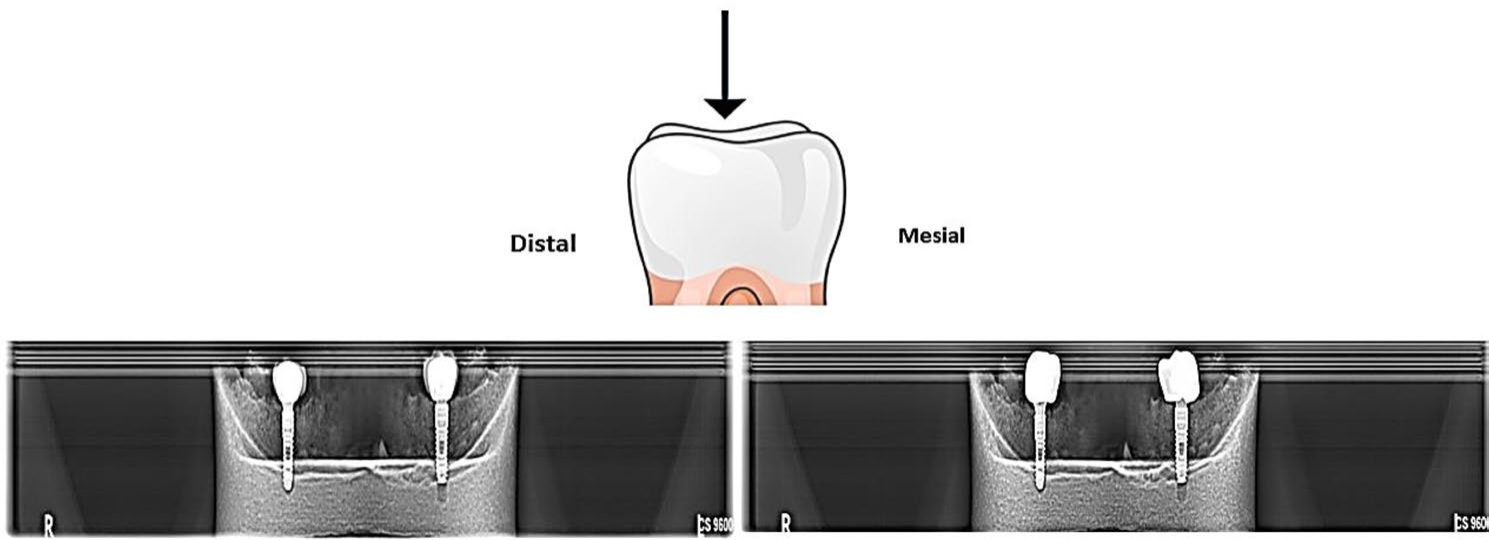
Scheme of control group perforation

For control groups (GIA and GIB), the OPG X-ray (Exposure:73 kV,8.0 mA,12.3s, Dose:122mGy.cm^2^)was used to localize the implant direction. The mesiodistal position was localized approximately by drawing a line from the end of the fixture, passing through the implant direction and the crown, and the existing line from the occlusal surface was marked as the implant axis and the distances from the proximal point mesially and distally to the localized current point of the line were measured. The recorded measurement from the OPG was used as a guide for locating the drilling site on the crown occlusal surface (Fig. 4).

**Fig. 5:**
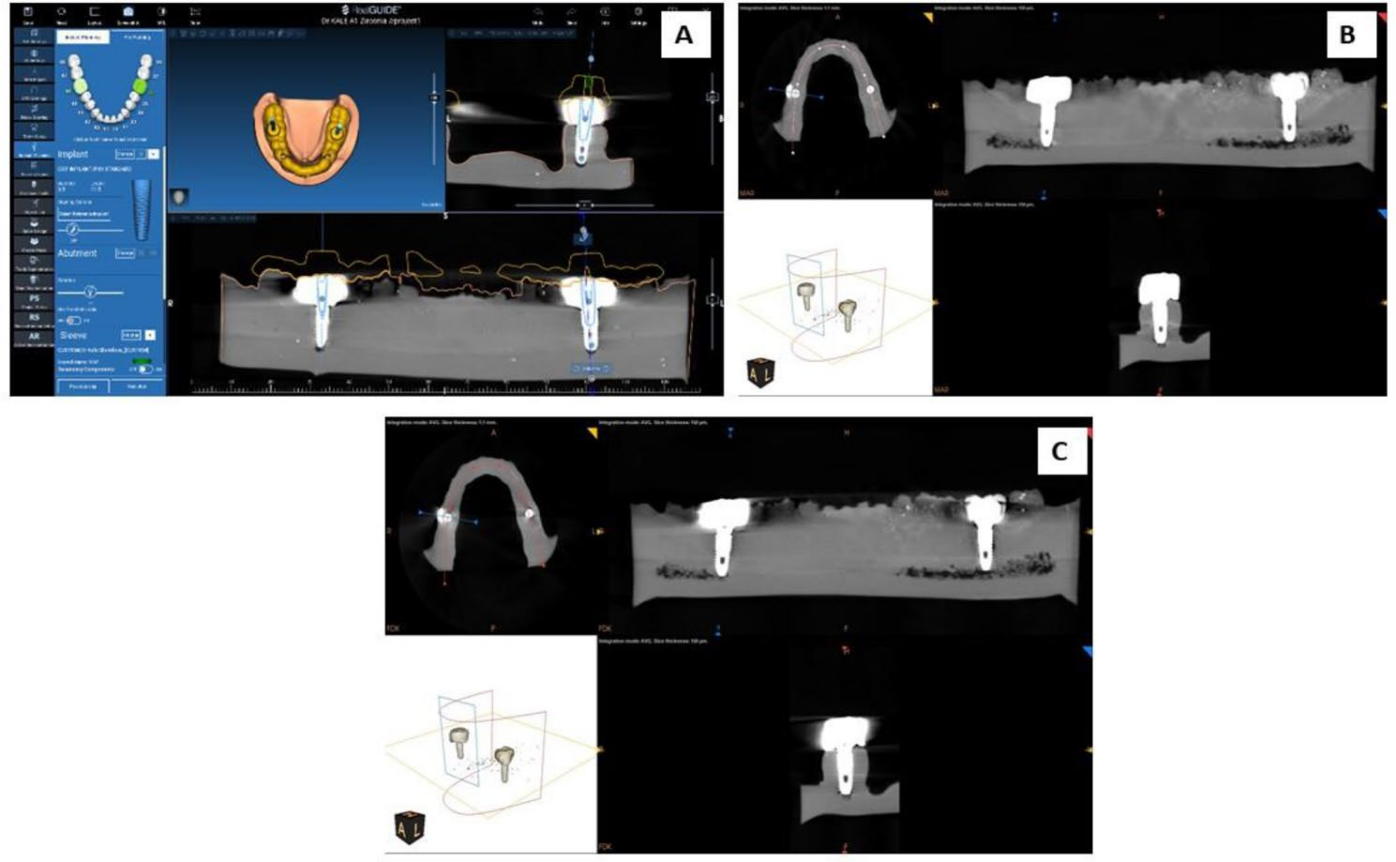
Explanation scheme of groups II and III. CBCT for samples (**A**), plane CBCT (**B**), and CBCT + MAR (**C**). CBCT: Cone beam computed tomography; MAR: Metal artifact reduction

Whereas for CBCT (Exposure:120KV, 6.3 mA,20s, Dose: 673mGy.cm^2^) (GIIA and GIIIA) and CBCT + MAR (GIIB and GIIIB), the drilling site was localized by drawing imaginary lines passing through the long axis of the fixture and continuing through the crown to the outside in the X, Y, and Z axes (Fig. 5).

**Fig. 6:**
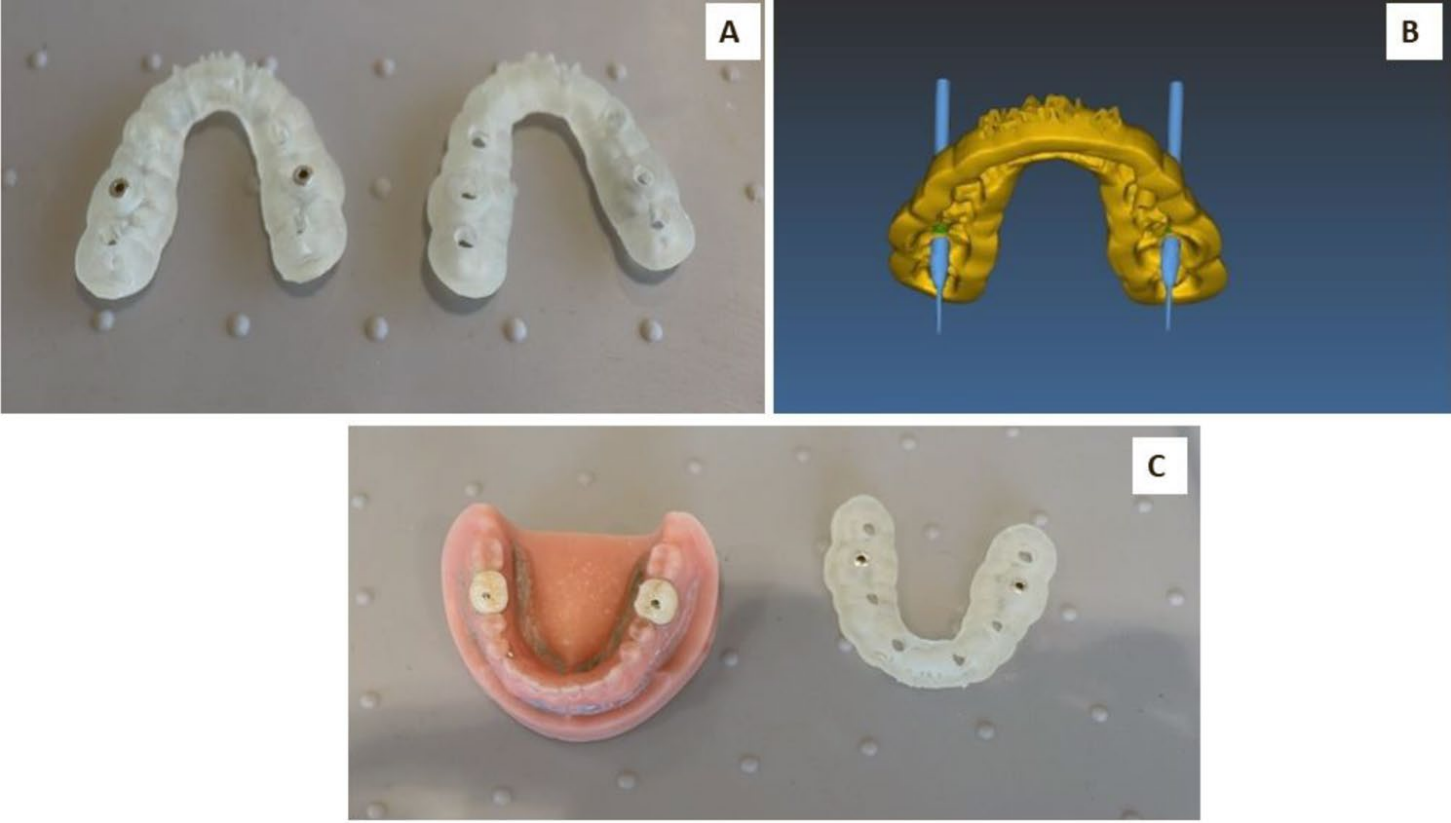
Construction of surgical guide steps with metal sleeve (**A**), designed perforation site (**B**), and crown after perforation (**C**)

SCANNER Carestream CS9600 was used to scan all models, and results were combined with CBCT results to fabricate a surgical guide (SprintRay pro 95 SG2 resin) that was designed with a hole on the occlusal surface at the emergence of the imaginary line in the X, Y, and Z axes. The hole size was adjusted to 2 mm diameter with metal sleeves (designed in real guide software version 5.1, metal lined printed in cobalt chrome by Ritton Laser printer) to stabilize the drilling burs (Fig. 6).

### Drilling stage procedure

The crowns were drilled using a Meisinger bur 881Z five coarse for zirconia (GIA, GIIA, and GIIB). In contrast, Meisinger bur HMG37RS coarse was used for CFM crowns (GIB, GIIIA, and GIIIB) by electrical high-speed handpieces at a constant speed with water air spray, with one drilling bur used for each implant. The drilling was performed through the metal sleeves (Fig. 6). The drilling was continuous until the bur reached the abutment.

#### Scanning electronic microscopy (SEM) investigation

**Fig. 7:**
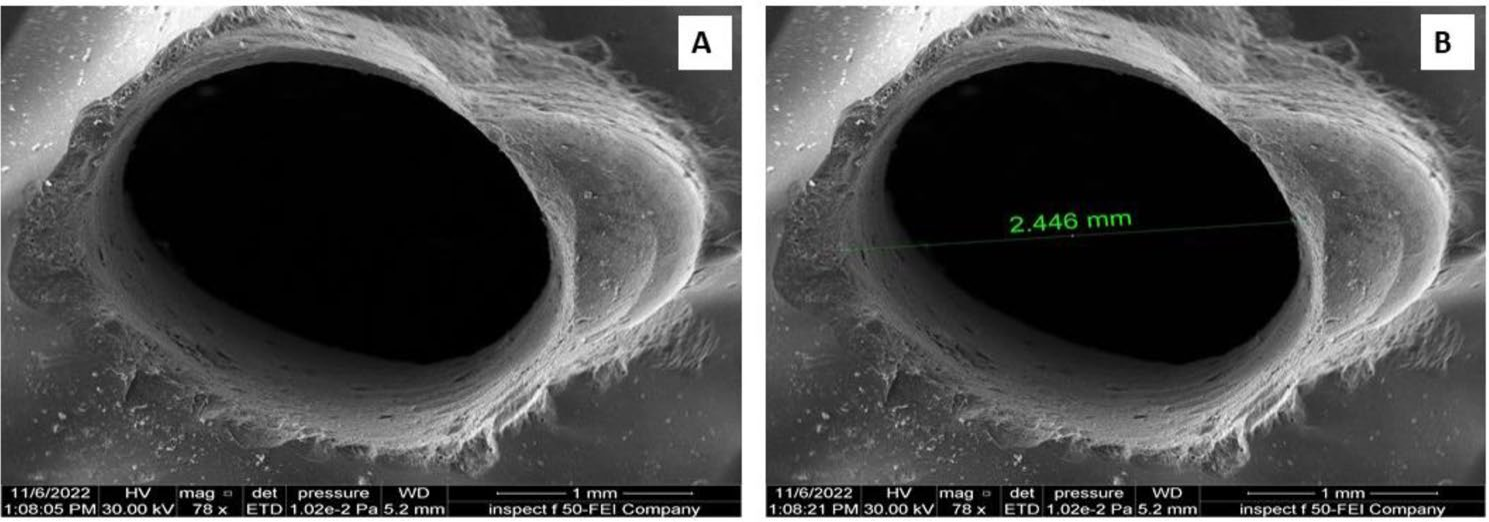
Shows crack (**A**) and diameter (**B**) of the crown hole using Scanning Electron Microscopy

The surfaces of crowns were observed by SEM **(**Inspect TM F50, Smart SCAN, Nav-Cam, FEI Company, USA) for cracking and chipping. Then, each crown’s chipping percentage was calculated (Fig. 7; Table 1).

**Table 1:** Crack and chipping by scanning electron microscopy

Variable	Status	Zirconia crown	Metal ceramic crown	p-value
		Number (%)		
Cracks	Yes	11 (18.3)	21 (35)	0.009**
	No	19 (31.7)	9 [15]	
Chipping	Yes	18 [30]	13 (21.7)	0.19
	No	12 [20]	17 (28.3)	

*: Highly significant difference using Chi-square test

### Micro-CT scan

**Fig. 8:**
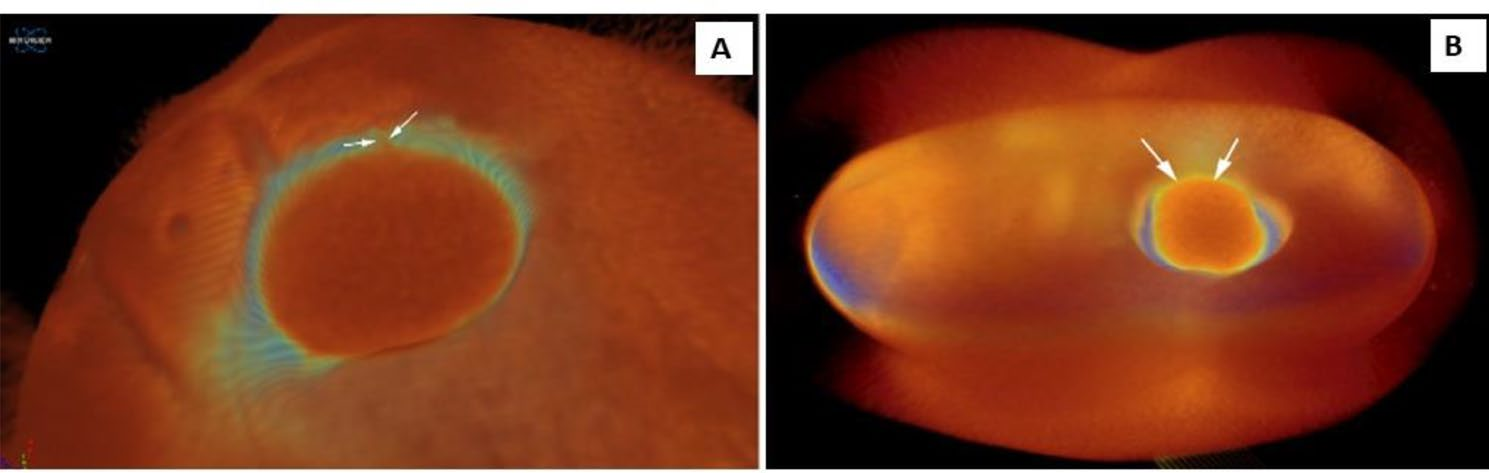
Micro-CT of the crowns indicating cracks (**A**) and chipping (**B**)

The interior surfaces of the SAH were scanned interiorly by Micro-CT (Skyscan 1275, Bruker Kontich, Belgium); the scanning process was set as 100 KVp, 100 Ma beam current 0.5 mm A1 filter, 15.1 mm pixel size, rotation at 0.5 steps for crack (0-no,1-yes), chipping (0-no,1-yes), and volume around the hole (mm^3^) (Fig. 8).

### Statistical analysis

Statistical Package for Social Science (SPSS, version 25) (IBM, Chicago, USA) was used for data analysis. Descriptive results were presented as means and standard deviation (SD) for quantitative data and as frequency (%) for categorical data. The chi-square test was used to compare data between groups, while Tukey’s (Dunn’s) test was used to compare significance levels between subgroups. P-values of < 0.05 were statistically significant, while *p* < 0.001 was set as a highly significant difference.

## Results

Regarding the effect of plane CBCT and CBCT + MAR on prepared crowns, there is a highly significant difference (*p* = 0.001) between zirconium crowns subgroups (G1A, GIIA, and GIIIA) and the CFM subgroups (*p* = 0.002) (GIB, GIIB, and GIIIB), but no significant difference between control groups and plane CBCT (GIA and GIIA) (GIB and GIIIB) (Table 2). According to the Shapiro-Wilk normality test, the data was not normally distributed for the diameter of the hole (*p* = 0.001), so Kruskal Wallis was used with Dunn’s test.

**Table 2:** Effect of plane CBCT and CBCT + MAR on prepared crowns

Diameter of the hole	Sub-group (No.=10)	Mean ± SD	Min-Max	Median (Q1-Q3)	Tukey’s test*	*p*-value
Zirconia crown	Control (GIA)	4.021 ± 0.511	3.07–4.08	3.96 (3.595–4.57)	a	0.001**
	Plane CBCT (GIIA)	2.911 ± 0.252	2.44–3.45	2.9 (2.825–3.06)	b	
	CBCT + MAR (GIIIA)	3.311 ± 0.549	2.56–4.65	3.185 (2.872-3.7)	bc	
Metal-ceramic crown	Control (GIB)	3.478 ± 0.5179	2.66–4.66	3.57 (3.06–3.845)	a	0.002**
	Plane CBCT (GIIB)	2.981 ± 0.319	2.55–3.9	2.96 (2.8-3.057)	b	
	CBCT + MAR (GIIIB)	3.524 ± 0.569	2.66–4.8	3.55 (2.94–3.945)	ca.	

*Results with the same letter do not show significant differences, **: Highly significant difference using Tukey’s-test

CBCT: Cone beam computed tomography; MAR: Metal artifact reduction

Significant differences between the zirconia crowns groups and CFM groups were found regarding the volume of chipping and cracking of SAH after drilling. The effect of the burs on the chipping volume in the zirconium crown groups in the mean, standard deviation, min and max, and median is more significant than in the CFM groups, with the results being effective (*p* = 0.04) (Table 3).

**Table 3:** Volume of chipping

Volume of chipping (mm3)	Number	Mean ± SD	Min-Max	Median	*p*-value
Zirconia crown	30	31.03 ± 13.14	10–56	35.0	0.04*
Metal-ceramic crown	30	24.5 ± 11.37	12–48	20.0	

*: Significant difference using Chi-square test

The crack and the chipping on the surface of the zirconium crown and CFM crowns were analyzed by SEM and Micro-CT. Comparison of the cracking of SAH showed significant differences between the zirconium crown and CFM restoration (*p* = 0.009). At the same time, for the chipping, there were no significant differences between zirconium and CFM restoration (*p* = 0.19) (Table 1).

## Discussion

Regarding the results of the present study, in the zirconium crowns and CFM restoration groups, there was better localization of the SAH, with the diameter of SAH in the CBCT plane, CBCT with MAR being smaller than the control group, where the localization was done by 2D OPG radiograph. These data represent that CBCT has a better effect on the localization of the screw of the implant as a tool for guiding to the precise location of the implant abutment screw hole. Thus, retrieval of the loosened cement-retained implant prostheses and the integrity of the prosthesis and implant abutment depend on the drilling technique, the accuracy of the drilling guide, and the existing access channel direction. Hence, with a metal sleeve (2 mm), the burs could accurately perforate the crowns without shifting from the original path made by the CBCT plane or CBCT + MAR, an imaginary arbitrary line extending from the implant to the crowns. If the SAH is outside the occlusal site, such as on a cusp tip area or labial surface, the retrieval and reuse of the loosened cement-retained implant prostheses may not be preferable. A new restoration should be fabricated to remove potential fractures, and the trend of the implant SAH should be sensibly assessed during the diagnosis, which agrees with the outcomes of Neshandar et al. and Asli et al. [23, 24], who also achieved better localization with the aid of CBCT. In the control group, regarding the localization by 2D X-ray OPG, the size of the SAH was significant and traumatized and compromised the strength of the crowns.

One of the benefits of this approach is the elimination of freehand drilling as in the control group, in which the SAH localization is only done in a mesiodistal direction that reduces the possibility of damaging the existing crown and the abutment. This procedure is particularly beneficial in dental clinics when there are no pre-cementation records of the patient. Also, the CBCT and 3D-printed drilling guide cost less than fabricating a new crown. In addition, the CBCT-driven drilling guide may produce a smaller access opening, which results in less damage than typical freehand drilling.

A selected small field of view of CBCT machine used to minimize patient exposure to radiation and limit the area of exposure the field of view to include 2 to 3 teeth [23], and avoiding unnecessary exposure to adjacent tissues, in addition, by adjusting patient positioning (tilting the chin). Supplementary use of personal security, up to 40% dosage reduction can be accomplished (thyroid collar) [25].

Substantial efforts have been made to establish MAR algorithms to decrease beam hardening effects and recover CBCT scan quality. Some CBCT machines can apply a pre- or post-acquisition MAR algorithm that is announced to reduce artefacts and enhance image quality. Pre- and post-acquisition MAR is based on standardizing voxel values by enhancing the reconstruction of the image and improving the contrast-to-noise ratio (CNR) to diminish the harmful impacts of artefacts [26], which agrees with our finding of significant differences between the control group and the groups on which CBCT with MAR was used. The MAR system used in both the zirconium crown group and the control group showed significant differences from the group in which MAR software was used to accurately position the SAH, which disagrees with Kamburoglu et al. [27] It can be concluded that MAR does not influence the diagnosis of periodontal and peri-implant defects on CBCT images, according to Bechara et al. [28] The inconsistency between objective and subjective assessments on the efficacy of MAR highlights the importance of evaluating possible factors that may compromise the action of this tool.

In some cases, the patient record or data are available, which aids the retrieval of the loosened cement-retained implant prostheses and presents a straightforward method for locating the SAH, which preserves the prosthesis and implant abutment, minimizing the dimension of SAH.

The surfaces of the crowns were examined by SEM for cracks and chipping of SAH since SEM would show the effect of the burs on the crown surface in terms of the existence, number and extent of cracks, as demonstrated in another study of cracking of the porcelain surface [29]. The interior surface of the SAH was examined by Micro-CT, which aided in a detailed examination of the volume of chipping and cracking of the SAH since it gives very accurate detail of the interior surfaces of the crowns [30], showing that the cracking in the zirconium crown group was less than in the CFM group, with a significant difference.

Furthermore, crown fracture or chipping and cracking may occur despite accurate localization of the screw access hole; other factors may contribute to this matter like the bur shape, surface texture, size and materials made from. The author suggested that more studies are required to investigate the effect of the mentioned factors above on the development of chipping and cracks for metal ceramic and zirconium restoration during screw access hole preparation.

Although this study was designed to simulate the actual clinical setting by providing CBCT radiographs and conducting the drilling sessions inside the oral cavities of phantom heads, the findings are limited by excluding patient factors. More comprehensive clinical studies are necessary to assess clinical proficiency, procedural duration, convenience, and satisfaction to confirm the results of the present study; besides that, the cost of the CBCT and the surgical guide the effect of ionizing radiation more chipping in the surface of the crown during drilling procedure are the main limitations of this study.

## Conclusions

CBCT alone or with MAR significantly improved the correctness of drilling the screw channel and diminished injury to the existing restoration and abutment reliefs in better localization of SAH in loosened implant abutment screws which in turn led to solving the problem of losing abutment without replacing the crown and jeopardizing the stability of the implant.

## Data Availability

The datasets used and analyzed during the current study are available from the corresponding author upon request.
